# Mechanisms linking school physical education policies to college students’ physical activity: an empirical test of a moderated mediation model

**DOI:** 10.3389/fpsyg.2026.1663134

**Published:** 2026-01-28

**Authors:** Ming Liu, Huanju Liu, Zhuzhu Qin, Yining Tao, Ruizhe Jiang, Yanxia Zhong, Wan Ye, Xinxin Ye

**Affiliations:** 1 Department of Public Physical and Art Education, Zhejiang University, Hangzhou, China; 2 Department of Medical Genetics, Naval Medical University, Shanghai, China; 3 Department of Orthopedics, People’s Hospital of Deyang City, Deyang, Sichuan, China; 4 Department of Global Public Health, Karolinska Institutet, Stockholm, Sweden; 5 Department of Sports Science, Zhejiang University, Hangzhou, Zhejiang, China; 6 Department of Nursing, Shijiazhuang Medical College, Shijiazhuang, Hebei, China; 7 Department of Nursing, Xiamen Medical College, Xiamen, Fujian, China

**Keywords:** gender difference, grit, physical activity, school physical education policies, time management ability

## Abstract

School physical education policies (SPEPs) promote college students’ physical activity and fitness, but the mechanisms linking policy to behavior are under-explored. This study explores how SPEPs influence physical activity, focusing on time management’s mediating role and grit’s moderating effect. A survey of 1,151 students from six universities used scales measuring attitudes, time management, grit, and physical activity. Results showed males scored higher in physical activity, time management, and grit. SPEPs indirectly influence physical activity through time management, with grit moderating this pathway. Recommendations include enhancing policy support, time management training, fostering positive psychological traits, and gender-sensitive interventions.

## 1 Introduction

Since insufficient physical activity among college students has emerged as a pressing public health concern, understanding—and ultimately reversing—this trend is critical for safeguarding young adults’ long-term health and fitness. The van Sluijs’s et al. (2021) Global Report on Physical Inactivity among Adolescents reveals that over 81% of adolescents fail to meet daily activity recommendations, with Chinese youth showing especially high sedentary rates and low exercise levels (Guthold et al., 2020). Upon entering university, students face a dramatic reduction in organized physical education, heightened academic pressures, and newly autonomous schedules—all of which exacerbate their “disconnect” from regular exercise (Zhu et al., 2023) and risk entrenching sedentary habits without timely intervention. This period thus constitutes a critical window where sedentary behaviors can become entrenched, making the university environment a pivotal setting for interventions designed to foster lasting healthy habits.

School Physical Education Policies (SPEPs) have thus gained prominence as a structural lever for promoting campus-wide physical activity (Liu et al., 2022; Chang et al., 2025). These policies blend “hardware” elements—curricula, facilities, and staffing—with “soft” regulations such as evaluation frameworks, incentives, and promotional campaigns (Wu et al., 2024). However, a well-documented “policy-behavior gap” persists; the mere existence of these policies often shows a weak and inconsistent correlation with students’ actual exercise participation (Hu et al., 2021). This well-documented “policy-behavior gap” suggests that the pathway from institutional policy to individual behavior is not direct. Instead, its effectiveness is likely mediated by key psychological resources and contingent upon individual differences (Feng et al., 2022).

To bridge this policy–behavior gap, we must unpack the psychological pathways through which SPEPs exert influence. The Social Ecological Model provides a valuable starting point, emphasizing that health behaviors emerge from multi-level interactions. Within this framework, policies at the outermost “macro” level shape the social environment but influence individual decisions only indirectly through resource availability, opportunity structures, and normative climate (Sallis et al., 2000; Maselli et al., 2018). A foundational step in this indirect pathway involves shaping behavioral intentions. Here, the Theory of Planned Behavior (TPB) is instructive, positing that intention—driven by attitude, subjective norm, and perceived behavioral control—is the immediate precursor to action (Ajzen, 1991). SPEPs can foster positive exercise intentions by shaping attitudes (e.g., valuing health), subjective norms (e.g., creating a campus culture of fitness), and bolstering students’ perceived control over exercise (e.g., ensuring access to resources) (Huang et al., 2023). While instrumental in forming intentions, the TPB framework is less well equipped to explain how these intentions are translated into sustained action, particularly when confronted with competing demands. This reveals the well-known “intention-behavior gap,” underscoring the need to focus on post-intentional, action-oriented mechanisms.

One key individual-level mediator of this policy effect is time management ability. According to Self-Regulation Theory, effective time management transforms intentions into concrete actions by helping individuals plan, prioritize, and allocate scarce resources (Zimmerman, 2000). University students’ competing demands—from coursework to social life—make time management crucial for sustaining extracurricular exercise (Aeon et al., 2021). Indeed, a growing body of research from 2023 and 2024 confirms that effective time management is a cornerstone of self-regulation, enabling students to navigate the complex interplay between academic workloads and personal health goals (Zarei Hajiabadi et al., 2023; O’Sullivan et al., 2024). We thus hypothesize that SPEPs foster physical activity in part by enhancing students’ time management skills. Specifically, we posit that policies mandating participation in PE classes or offering structured athletic programs introduce fixed, non-negotiable commitments into a student’s otherwise flexible schedule. To successfully integrate these required activities, students are compelled to engage in more deliberate planning, prioritization, and scheduling—the core components of effective time management. This process of actively navigating policy-driven constraints can enhance their general time management capacity, which in turn facilitates the consistent allocation of time for physical activity.

Yet even a strong mediating role for time management may not play out uniformly. Here, the trait of grit—a stable disposition reflecting sustained effort and passion toward long-term goals—can act as a moderator. Grit predicts consistent exercise participation, training adherence, and exercise persistence under challenging conditions (Duckworth et al., 2007; Rutberg et al., 2020; Dai et al., 2025). Specifically, recent studies highlight that grit is crucial for maintaining long-term exercise regimens, particularly when facing academic stress or scheduling conflicts that commonly derail less determined individuals (Tao et al., 2024; Hu et al., 2025). The moderating role of grit can be understood through the lens of Self-Determination Theory, which emphasizes the internalization of external regulations for self-directed behavior (Ryan and Deci, 2000; Dunston et al., 2022). First, grit likely strengthens the link between SPEPs and time management. We argue that gritty individuals are more prone to internalize the policy’s objectives, reframing its logistical demands as opportunities for growth. This proactive mindset enhances their development of time management skills. Second, grit should amplify the influence of time management on physical activity. While good time management creates opportunities for exercise, grit provides the crucial perseverance to act on these plans, especially when facing fatigue or stress. Consequently, we expect that grit will amplify (1) the impact of SPEPs on the development of time management ability and (2) the effect of that ability on actual exercise behavior, producing a moderated-mediation (dual-pathway) model.

**Figure 1:**
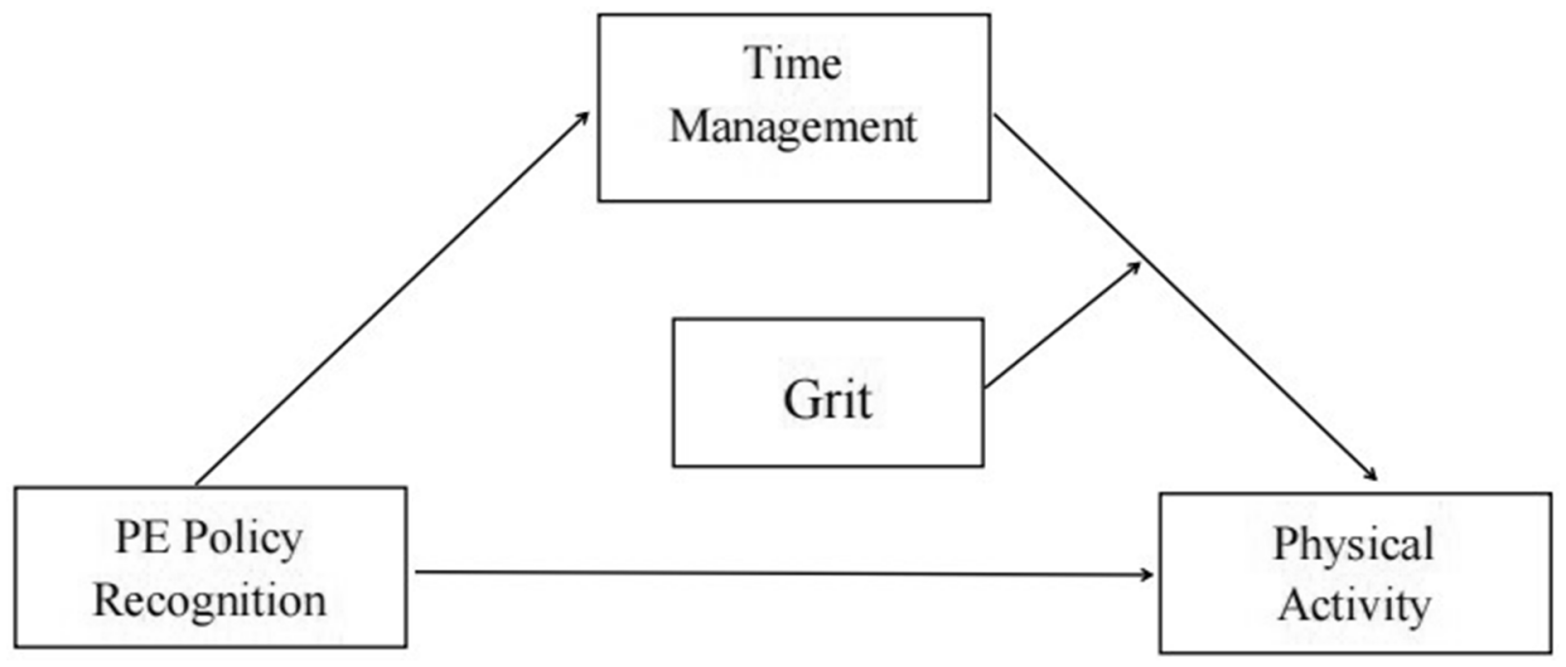
The moderated mediation model.

Although the challenge of translating school physical education policies into consistent student physical activity is a recurrent theme in the literature, existing explanatory models often fail to account for the complex interplay between practical skills and personality traits. Consequently, they provide an incomplete picture by inadequately clarifying the process of policy influence and the conditions under which it is most effective. The present study addresses this gap by proposing and testing a moderated-mediation framework (Figure 1) designed to answer three core questions: (1) To what extent do SPEPs directly predict college students’ physical activity? (2) Does time management ability mediate the SPEP–physical activity relationship? (3) Does grit strengthen either (or both) stages of this indirect pathway? The primary contribution of our study, therefore, is to test an integrated model that specifies how institutional support is converted into behavior (via the mechanism of time management) while simultaneously identifying for whom this pathway is most potent (contingent on the individual’s level of grit). By validating this framework, our research offers a more granular theoretical understanding that accounts for student heterogeneity and provides a foundation for developing targeted, rather than uniform, interventions designed to foster sustained physical activity.

## 2 Materials and methods

### 2.1 Study population and sampling method

This study adopted a stratified random sampling method, with undergraduate students as the target population. Universities were selected from Hangzhou and Nanjing, two major higher-education hubs in eastern China. These cities are characterized by comparable economic development levels, dense distributions of comprehensive universities, and similar governance frameworks for higher education and school physical education policy implementation.

At the same time, modest institutional variation exists between universities in the two cities in terms of policy implementation practices, allowing examination of policy-related mechanisms under relatively comparable yet non-identical regional contexts. Three universities were randomly selected from each city to ensure sample diversity and enhance the representativeness and external validity of the findings. The survey was conducted over a span of 10 days, from October 15 to 25, 2022.

### 2.2 Recruitment

Undergraduate students were selected as research subjects from a total of six universities in Hangzhou and Nanjing. Inclusion criteria were: (a) full-time undergraduate enrollment, (b) current registration at one of the sampled universities, and (c) voluntary participation with informed consent. Exclusion criteria included questionnaires with more than 25% missing responses or response patterns indicating inattentive completion. The research team relied on each university’s teaching management platform and student community network to collect data through an online questionnaire on the Wenjuanxing platform. Questionnaire links were distributed anonymously and spread with the help of class committee members, student union members, and interest club members in academic affairs systems and social media groups to expand coverage and improve response rates. To ensure questionnaire quality and recovery efficiency, this study also collaborated with some course instructors to encourage students to complete the questionnaire collectively before the end of class. All participants received an informed consent form before filling out the questionnaire, clarifying the research purpose, data confidentiality measures, and the principle of voluntary participation.

### 2.3 Training and administration

Before the questionnaire survey, all managerial staff received standardized training to ensure procedural consistency throughout the research process. Research subjects independently completed the questionnaire on their mobile phones, and then the research team systematically collected the feedback. Before data collection, each participant signed an informed consent form confirming their voluntary participation and understanding of the research. Participants were required to read a written information sheet before answering, which outlined the research purpose, data management and storage procedures, and the core ethical principles followed in the study, namely anonymity, voluntary participation, and confidentiality. Participants were also clearly informed of their right to withdraw from the study or stop completing the questionnaire at any stage.

### 2.4 Data collection

The questionnaire was divided into two parts: one part was the basic information of the survey subjects, including demographic information such as gender and age; the other part was modules such as SPEPs and atmosphere, sports motivation, time management, and positive character traits. Before the formal survey, a pre-test was conducted in one university using a face-to-face filling form, with 50 questionnaires distributed and 49 valid questionnaires recovered, with an effective rate of 98%. During this process, items with unclear semantics or difficult to understand were adjusted. A total of 1,200 questionnaires were officially distributed, and 1,151 valid questionnaires were recovered, with an effective recovery rate of 95.8%. To test for potential common method bias, procedural control methods and Harman’s single-factor method were used. In the procedural control method, the importance of conducting the survey “for scientific purposes only” was emphasized, achieved by separating the measurement of time and space from other strategies and particularly emphasizing the anonymous and voluntary nature of the survey. Data were collected by immediate completion and return to ensure better control of potential common method bias. The results of exploratory factor analysis without rotation extracted 13 factors with eigenvalues > 1, and the maximum factor variance explained was 27.68%, less than the critical standard of 40%. The results indicated that there was no serious common method bias among the variables, confirming that the common method bias in this study was within an acceptable range.

### 2.5 Measuring instruments

#### 2.5.1 School physical education policy attitude scale

To assess students’ perceptions of school physical education policy, we employed the 6-item School Physical Education Policy Attitude Scale (Chen and Gu, 2018). As a short instrument, the scale was treated as a unidimensional measure and a total score was computed. Based on the policy implementation theory, the items were designed to cover five key aspects of attitudes toward school physical education policy—content identification, implementation identification, effect identification, satisfaction, and behavioral intention—as content domains rather than separate subscales, with a total of 6 items. It uses a Likert 5-point scale, where higher scores indicate higher recognition. The reliability and validity test of the scale showed high internal consistency (Cronbach’s *α* = 0.876), and the KMO value was 0.917, indicating that the data were suitable for structural analysis, and the scale had good structural validity, suitable for the follow-up analysis of this study’s samples.

#### 2.5.2 Time management scale

This study used the Chinese revised version of the Adolescent Time Management Disposition Scale (Huang and Zhang, 2001) to evaluate individuals’ time management traits. The scale has a total of 44 items, covering three dimensions: time value, time monitoring, and time efficiency, using a Likert 5-point scale scoring, where higher scores indicate stronger time management tendencies. The reliability and validity test of this scale showed good internal consistency (Cronbach’s *α* = 0.953), and the KMO value was 0.970, supporting its structural validity and suitable for further analysis of this study’s samples.

#### 2.5.3 Grit scale

This study used the short-form grit scale (Grit-S) to evaluate college students’ persistence and frustration resistance in the pursuit of long-term goals (Duckworth and Quinn, 2009). The scale contains 8 items, belonging to two dimensions: goal consistency, reflecting whether individuals maintain stable interests and aspirations over a long period; and effort persistence, measuring individuals’ tendency to continue efforts in the face of difficulties. Each item uses a 5-point Likert scale, with higher scores indicating higher grit levels. The reliability and validity test of this scale in this study showed acceptable internal consistency (Cronbach’s *α* = 0.678), and KMO = 0.736, supporting its structural validity and suitable for this study’s analysis. We administered a previously validated Chinese version of the Grit-S rather than conducting a new translation or cultural adaptation. Prior psychometric research has supported the reliability, two-factor structure, and validity of the Chinese Grit-S in Chinese adolescents (Li et al., 2016).

#### 2.5.4 Physical activity rating scale

This study used the Physical Activity Rating Scale (PARS-3) to measure college students’ physical activity levels (Liu M. et al., 2024). The scale evaluates individuals’ physical exercise conditions from three dimensions: exercise intensity, frequency, and duration, with high internal consistency (Cronbach’s α = 0.805) and a KMO value of 0.846, having good reliability and validity, and has been widely used in the study of exercise behavior among Chinese adolescents and college students. The scale has three parts: exercise intensity refers to the degree of exertion during exercise, including “What is the intensity of your physical exercise?”; exercise frequency refers to the frequency of engaging in physical activity within a week, including “How many times do you engage in physical exercise per week?”; exercise duration refers to the duration of each exercise, including “How long does each of your physical exercises last?” Each item uses a 1–5 five-level scoring standard, where lower to higher scores represent weaker to stronger exercise levels. The final physical activity grade score is calculated according to the formula “intensity score × (duration score − 1) × frequency score,” with a score range of 0–100 points. According to the score results, it is divided into three categories of physical activity grades: low intensity (≤19 points), moderate intensity (20–42 points), and high intensity (≥43 points), used to measure the differences in college students’ individual physical exercise levels.

### 2.6 Data processing and analysis

All statistical analyses were performed using IBM SPSS Statistics (Version 26.0; IBM Corp., Armonk, NY, USA). First, descriptive statistics were calculated to examine the distribution and central tendencies of key variables. Independent samples t-tests were conducted to analyze gender differences in school physical education policy recognition, time management ability, grit, and physical activity levels. Spearman’s correlation analysis was conducted to examine bivariate associations among the main study variables. To further explore the mechanisms through which school physical education policies influence college students’ physical activity behavior, moderated mediation analysis was performed using PROCESS macro (Model 14) developed by Hayes (Version 4.1). Because PROCESS is a regression-based method rather than a structural equation modeling approach, it yields unstandardized coefficients (B), SE, and bootstrapped confidence intervals but does not provide SEM-style model fit indices. Accordingly, the present study adopts a path-analytic framework based on composite variables, rather than a latent-variable structural equation model with an explicit measurement component. In this model, school physical education policies served as the independent variable (X), physical activity behavior as the dependent variable (Y), and time management ability as the mediator (M), with grit serving as the moderator (W). Specifically, grit was specified to moderate the association between time management (M) and physical activity (Y) (i.e., second-stage moderation), and the interaction term (M × W) was included in the outcome model. The significance of the indirect effect was tested using bias-corrected bootstrapping (5,000 samples) with 95% confidence intervals. An indirect effect was considered statistically significant if the CI did not include zero. The moderated mediation effect was evaluated using the index of moderated mediation and its bootstrap confidence interval.

## 3 Results

### 3.1 General characteristics of research subjects and between-group comparisons of physical activity

A total of 1,200 questionnaires were distributed. The screening criterion was “more than 25% missing responses,” and finally, 1,151 valid data were obtained, with an effective recovery rate of 95.8%. Key results from the analysis of physical activity in the college student group showed that males (*n* = 735, 63.9%) had a significantly higher mean value (*M* = 136.58, SD = 31.34) than females (*n* = 416, 36.1%, *M* = 119.60, SD = 29.81), with a statistically significant difference (*p* < 0.001). There were no statistical differences in age, grade, major, household registration location, and whether being an only child (*p* > 0.05). The results are shown in Table 1.

**Table 1:** Demographic characteristics of study participants and differences in physical activity (*n* = 1,151).

Variable	Group	*N* (%)	Physical activity (*M* ± SD)	*t/F*	*p*
Gender	Male	735 (63.9)	136.58 ± 31.34	8.985	<0.001
	Female	416 (36.1)	119.60 ± 29.81		
Age	19	424 (36.9)	129.31 ± 32.42	1.485	0.217
	20	367 (31.9)	130.28 ± 26.83		
	21	231 (20.1)	134.14 ± 31.67		
	22	129 (11.2)	133.58 ± 25.75		
Grade	Freshman	424 (36.9)	129.31 ± 32.42	1.485	0.217
	Sophomore	367 (31.9)	130.28 ± 26.83		
	Junior	231 (20.1)	134.14 ± 31.67		
	Senior	129 (11.2)	133.58 ± 25.75		
Discipline	Engineering	549 (47.7)	134.40 ± 32.02	4.889	0.082
	Science	202 (17.2)	128.18 ± 33.26		
	Humanities	216 (18.8)	124.52 ± 28.85		
	Medical	118 (10.3)	126.06 ± 30.56		
	Agricultural	66 (5.7)	131.70 ± 33.86		
Registration	Urban	809 (70.3)	130.14 ± 32.23	−0.499	0.618
	Rural	342 (29.7)	131.17 ± 30.97		
Only child	Yes	598 (52.0)	130.59 ± 32.47	0.166	0.868
	No	533 (48.0)	130.28 ± 31.19		

M, mean; SD, standard deviation. Group differences were tested using independent-samples *t*-tests or one-way ANOVA, as appropriate.

### 3.2 Relationship between sports policy attitudes and physical activity of college students of different genders

Male students had significantly higher scores than female students in SPEP recognition, time management ability, grit, and physical activity (*p* < 0.05), as shown in Table 2. Specifically, male students scored higher than female students in SPEP recognition (*t* = 2.585, *p* = 0.010, Cohen’s d = 0.159). Although the effect size was small, it indicated that males had a certain advantage in policy recognition; the gender difference in time management was more significant (*t* = 4.473, *p* < 0.001, Cohen’s d = 0.294), indicating that male students may have higher efficiency and planning in daily time arrangement and self-management. In terms of grit, males also significantly higher than females (*t* = 4.344, *p* < 0.001, Cohen’s d = 0.273), reflecting that males showed stronger willpower in the face of challenges and goal persistence. In the key indicator of physical activity, males scored much higher than females (*t* = 7.640, *p* < 0.001, Cohen’s d = 0.469), with a medium effect size, highlighting the significant gender difference in actual sports participation behavior.

**Table 2:** Gender differences in key study variables.

Variable	Male (*n* = 735)	Female (*n* = 416)	*t*	*p*	Cohen’s d
SPEP recognition	40.794 ± 6.514	39.791 ± 5.985	2.585	0.010^*^	0.16
Time management ability	161.265 ± 27.330	154.158 ± 23.131	4.473	<0.001^***^	0.29
Grit	28.345 ± 4.829	27.091 ± 4.144	4.344	<0.001^***^	0.27
Physical activity	33.394 ± 26.484	21.846 ± 20.968	7.640	<0.001^***^	0.47

Values are presented as mean ± SD. Group differences were tested using independent-samples *t*-tests. Effect sizes were estimated using Cohen’s d. SPEP Recognition: School Physical Education Policy Recognition. *p < 0.05; ***p < 0.001.

### 3.3 Score status and correlation analysis of sports policy, time management, grit, and physical exercise

According to the correlation matrix (Table 3), all variables showed significant positive correlations, providing preliminary support for the subsequent mediation effect and moderated mediation effect analysis. Specifically, the correlation coefficient between time management and grit was the highest (*r* = 0.497, *p* < 0.001), indicating that good time management ability may be closely related to higher grit levels; both are regarded as important psychological resources affecting sports behavior. There was also a moderate positive correlation between SPEP and time management (*r* = 0.312, *p* < 0.001), indicating that the higher students’ recognition of sports policies, the better their time management level, possibly due to the potential guiding role of policies in regulating daily behavior and stimulating self-discipline. In addition, the correlation coefficient between grit and physical activity was *r* = 0.067 (*p* < 0.05). Although the effect size was small, it was still statistically significant, suggesting that grit traits may affect students’ exercise persistence to a certain extent. Similarly, the positive correlation between time management and physical activity (*r* = 0.121, *p* < 0.001) also showed that the ability to manage time has a positive effect on increasing sports participation frequency. The correlation coefficient between SPEP and physical activity was *r* = 0.073 (*p* < 0.05). Although the correlation degree was low, its significance still indicated that policy perception had a basic impact on behavior.

**Table 3:** Descriptive and correlation statistics of each variable (*n* = 1,151).

Variable	Mean	*SD*	1	2	3	4
1. SPEP recognition	40.43	6.34	1			
2. Time management ability	158.69	26.11	0.312^***^	1		
3. Grit	27.88	4.63	0.210^***^	0.497^***^	1	
4. Physical activity	29.22	25.24	0.073^*^	0.121^***^	0.067^*^	1

Correlations are Spearman’s rho and are shown below the diagonal. SPEP Recognition, School Physical Education Policy Recognition. *p < 0.05; ***p < 0.001.

### 3.4 Analysis of the influence pathway of sports policy on physical activity

To test the influence mechanism of SPEPs on college students’ physical activity, this study used a mediation effect analysis method to construct a path model with time management as the mediating variable. The analysis results are shown in Table 4: (1) The total effect of sports policy on physical activity reached a significant level (*B* = 0.224, *p* = 0.050), indicating that students with higher policy recognition had higher physical activity levels, supporting the positive impact of sports policies on college students’ sports participation. (2) After introducing the mediating variable, the direct effect was no longer significant (*B* = 0.122, *p* = 0.309), while the indirect effect path was significant, indicating that time management played a key mediating role between sports policy and exercise behavior. Specifically, sports policy could significantly predict the improvement of time management ability (*B* = 1.251, *p* < 0.001), and time management significantly and positively affected physical activity (*B* = 0.082, *p* = 0.006), forming a significant indirect path between the two [*B* = 0.102, 95% CI (0.018, 0.200)]. (3) Overall, the influence of sports policy on physical activity was mainly indirectly realized through the mediating variable of “time management.” Although the “direct path” was not significant, the “indirect path” was clear and the effect was stable, indicating that time management ability played a bridging role in the process of policy promotion and behavior transformation.

**Table 4:** Regression-based mediation analysis of the association between SPEP recognition and physical activity.

Path	*B*	*SE*	*t*	*p*	LLCI	ULCI
SPEP recognition → time management ability (a)	1.285	0.115	10.88	<0.001***	1.026	1.477
Time management ability → physical activity (b)	0.090	0.029	2.78	0.006*	0.024	0.139
SPEP recognition → physical activity	0.163	0.123	1.02	0.309	−0.113	0.358
Indirect effect (a × b)	0.126	—	—	—	0.018	0.200
Total effect (c)	0.224	0.115	1.96	0.050	−0.001	0.450

Indirect effects were tested using bias-corrected bootstrapping with 5,000 resamples. Values in brackets represent the 95% bootstrap confidence intervals. The mediator model (time management ability) explained 9.75% of the variance (*R*^2^ = 0.0975, *F* = 124.16, *p* < 0.001), and the outcome model (physical activity) explained 2.22% of the variance (*R*^2^ = 0.0222, *F* = 6.51, *p* < 0.001). *p < 0.05; ***p < 0.001.

The mediation model explained 11.2% of the variance in time management (*R*^2^ = 0.0975) and 6.5% of the variance in physical activity (*R*^2^ = 0.022). The indirect effect (*B* = 0.102) accounted for 45.5% of the total effect, indicating a medium effect size according to conventional benchmarks.

### 3.5 Moderating effect of grit on time management and physical activity

To further explore the moderating effect of grit in the process of SPEPs influencing physical activity through time management, this study constructed a moderated mediation model and tested the interaction effect through two-stage regression analysis. The analysis results are shown in Table 5: (1) In the first path, SPEP had a significant positive predictive effect on time management ability (*B* = 1.251, *p* < 0.001), indicating that the higher the recognition of sports policies, the stronger the students’ time management ability. Gender also reached a significant level in this path (*B* = −5.581, *p* < 0.001), showing that female students’ overall time management ability was lower than that of male students. (2) In the second path, time management had a significant positive predictive effect on physical activity (*B* = 0.090, *p* = 0.005), once again verifying the key role of time management in promoting sports participation behavior.

**Table 5:** Moderating effect of grit on the association between time management ability and physical activity.

Predictor	Model 1: time management ability (M)				Model 2: physical activity (Y)			
	*B*	*SE*	*t*	95% CI	*B*	*SE*	*t*	95% CI
Gender	−5.58	1.52	−3.86	(−8.83, −2.87)	−10.65	1.524	−6.95	(−13.64, −7.66)
SPEP recognition	1.25	0.12	10.88^***^	(1.03, 1.48)	0.13	0.120	1.11^***^	(−0.10, 0.37)
Time management ability	—	—	—	—	0.09	0.033	2.74^***^	(0.03, 0.16)
Grit	—	—	—	—	−0.10	0.182	−0.53^***^	(−0.45, 0.26)
Time management ability × grit	—	—	—	—	0.01	0.004	2.28^***^	(0.001, 0.02)

Unstandardized coefficients are reported. Confidence intervals represent 95% confidence intervals. Model statistics: Model 1 (time management ability): *R*^2^ = 0.109, *F* = 70.26, *p* < 0.001; Model 2 (physical activity): *R*^2^ = 0.062, *F* = 15.19, *p* < 0.001. *p < 0.05; ***p < 0.001.

**Figure 2:**
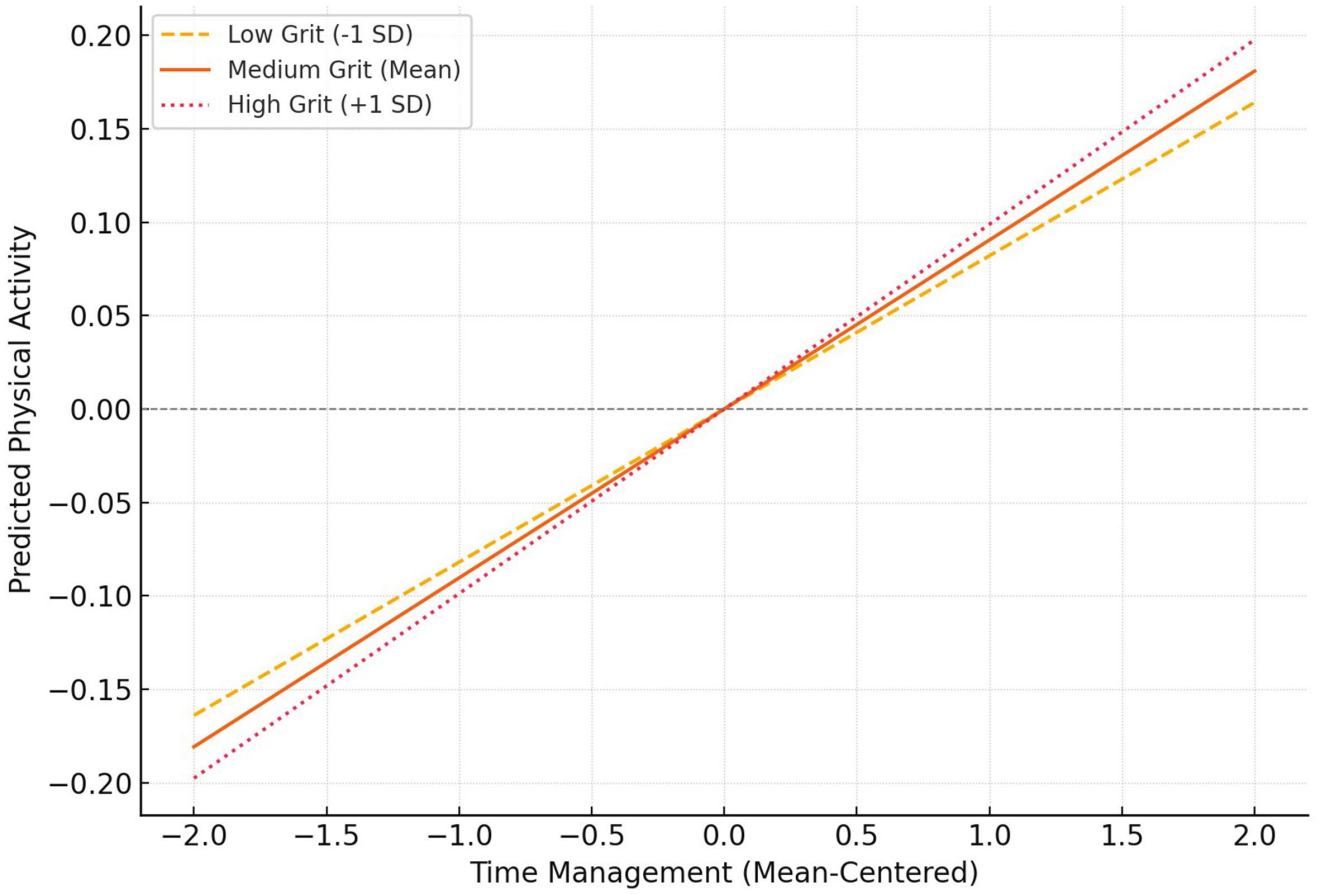
Moderating effect of grit on the relationship between time management and physical activity.

At the same time, the main effect of grit on physical activity was not significant (*B* = −0.096, *p* = 0.598), but it showed a significant moderating effect in its interaction with time management (*B* = 0.008, *p* = 0.023), indicating that grit could enhance the positive impact of time management on physical activity. Specifically, as the grit level increased, the promoting effect of good time management ability on physical activity became more significant. (3) In terms of model fit, both regression equations reached statistical significance (*F* = 70.263, *F* = 15.185, *p* < 0.001), and the model had good explanatory power (*R*^2^ = 0.109, *R*^2^ = 0.062), indicating that the model structure had a certain degree of stability and explanatory power. Grit played an important moderating role in the path of sports policy influencing physical activity through time management, that is, when students had higher levels of grit, their exercise behavior based on time management would be more stable and persistent (Figure 2).

## 4 Discussion

This study examined how awareness of SPEPs influences college students’ physical activity, with a focus on the mediating role of time management skills and the moderating effect of grit. Our findings indicate that greater policy awareness is linked to higher levels of physical activity, primarily through improved time management. Furthermore, the strength of the indirect pathway through time management was amplified for students with higher levels of grit. Taken together, these results offer a more nuanced explanation for the well-documented “policy-behavior gap,” suggesting that the link between institutional policies and individual behavior is channeled through the development of self-regulatory skills and is contingent upon key psychological dispositions.

We observed significant gender differences across key psychological and behavioral variables. Male students reported higher scores in PE policy awareness, time management ability, grit, and overall physical activity, with the largest disparity in exercise levels—an effect of medium magnitude. These results are consistent with prior research showing that cultural norms and societal expectations often encourage males to participate in competitive, vigorous sports, while females may feel constrained by stereotypes emphasizing gentleness and appearance concerns (McCarthy and Warne, 2022; Yu et al., 2024). While these sociocultural factors provide a partial explanation, our model suggests the gender gap is also rooted in a combination of structural and psychological factors. Furthermore, beyond these sociocultural factors, a recent study attributes this gender gap to more tangible, structural barriers, such as a lack of female-centric activities and safety concerns in campus facilities (Prieto-González and Alkouatli, 2025). Factors such as anxiety about appearance and fewer peer support networks can further limit women’s engagement in physical activities. In our moderated mediation model, gender acted as a significant covariate, influencing both time management and activity levels. These results suggest that policy interventions should be gender-sensitive, tailoring resource allocation, curriculum design, and evaluation methods to address the unique barriers faced by female students (Liu L. et al., 2024). This finding underscores that policy interventions must be explicitly gender-sensitive and address these concrete structural and environmental barriers to be truly equitable and effective.

Consistent with Self-Regulation Theory (Zimmerman, 2000), time management emerged as a key mediator in the relationship between PE policy awareness and physical activity. This finding helps to bridge the “policy-behavior gap” by illustrating a specific mechanism. The strong association suggests that when students perceive policies as requiring them to integrate structured activities into their schedules, they may be prompted to engage in more deliberate planning and prioritization—a behavioral response to environmental demands rather than a change in underlying cognitive ability. This echoes recent intervention studies where training in planning and scheduling skills led to significant and sustained increases in physical activity among young adults, confirming time management as a potent and malleable behavioral lever (Cao and Jiang, 2025). As evidenced by cross-cultural research, physical activity mitigates anxiety and sleep disturbances in youth (Solmaz et al., 2025), highlighting its universal benefits beyond immediate behavioral outcomes. This interpretation provides a compelling illustration of the Social Ecological Model in action (Sallis et al., 2000), demonstrating how a macro-level factor (policy awareness) can be associated with an individual-level self-regulatory skill (time management), which in turn is linked to a health behavior. Our study contributes by identifying time management not just as a predictor of healthy behaviors (Knowlden and Naher, 2023), but as a potential conduit through which institutional structures are associated with individual self-regulation. To maximize impact, future college sports policies should emphasize the development of soft skills—such as time management—by creating a comprehensive support system. Integrating curriculum planning, life-scheduling workshops, and incentive mechanisms can facilitate the transition from “policy guidance” to “behavioral cultivation.”

Further analysis revealed that grit significantly moderates the effect of time management on physical activity. Specifically, the interaction term between time management and grit was statistically significant, indicating that students with higher grit levels gain greater benefit from effective time management. In line with Self-Determination Theory (Xu et al., 2025), grit functions as an intrinsic motivational resource that sustains effort in the face of obstacles—academic pressures, fatigue, or lack of immediate rewards. This effect can be conceptualized through the two dimensions of grit: perseverance of effort and consistency of interest. Higher grit individuals appear better equipped to navigate the challenges of student life, as they report fewer perceived barriers to exercise (e.g., lack of time) and demonstrate greater psychological resilience against academic stress that might otherwise disrupt fitness routines (Yu et al., 2024; Zhang et al., 2024; Fu et al., 2025). In contrast, consistency of interest may explain why these individuals are more motivated to engage with and leverage SPEPs as valuable resources in the first place. This demonstrates that grit’s role is not merely that of a direct predictor of exercise, but can also function as a catalyst that amplifies the conversion of planning (time management) into action (physical activity), offering a more sophisticated understanding of its function in self-regulated behavior.

In conclusion, School Physical Education Policies (SPEPs) can promote college students’ physical activity, but their effectiveness is channeled through students’ enhanced time management skills and amplified by their individual grit. This implies that policy must be supported by targeted, operational interventions. For instance, to bolster the mediating role of time management, universities should offer practical, credit-bearing workshops that teach students specific strategies like time-blocking for exercise (Li et al., 2025). Similarly, to cultivate grit, building on emerging practices where institutions embed positive psychology modules into sports curricula (Huang and Long, 2025); we recommend operationalizing a four-stage developmental model, comprising: (1) goal setting, (2) process feedback, (3) willpower training, and (4) reflection. This model can be put into practice by guiding students to create long-term SMART fitness plans (goal setting) and use structured journaling to reflect on overcoming setbacks (reflection). Furthermore, a truly gender-sensitive approach requires concrete actions, such as diversifying activity offerings to include more female-centric options (e.g., yoga, dance) and making tangible improvements to facility safety. By integrating these specific, evidence-based interventions—from skill-building workshops to psychologically informed curriculum design—universities can translate policy into a powerful framework that fosters sustainable, intrinsic, and high-quality student participation in physical activity.

### 4.1 Limitations and future directions

Despite offering valuable insights, this study has several limitations that should guide future research. First, our study is constrained by its research design and data source. Specifically, because all data were collected at a single point in time, we cannot rule out the possibility of reverse causality—for instance, that strong time management skills may lead to greater awareness of SPEPs, rather than the other way around. Consequently, future longitudinal studies are needed to establish temporal precedence, while experimental designs, such as a randomized controlled trial (RCT) testing a time management intervention, would be required to firmly establish causality.

Second, there are limitations related to variable measurement and operationalization. Our independent variable was students’ subjective perception of SPEPs, not objective policy metrics, which limits direct evaluation of specific policy components. Additionally, the modest internal consistency of the Short Grit Scale may have introduced measurement error, potentially attenuating its moderating effect. Future studies could bridge these gaps by combining survey data with objective institutional policy data and using more robust psychological scales.

Third, our statistical model has inherent limitations. The analysis was conducted using a regression-based path model with composite variables, which does not allow for the evaluation of a full measurement model as in SEM. While procedural controls were used, common-method bias cannot be entirely discounted. Critically, the model’s modest explanatory power for physical activity indicates that our chosen pathway, though significant, accounts for only a small fraction of the variance in this complex behavior.

Finally, the sample’s generalizability is limited. Data were collected from six universities in two major cities in eastern China, so the findings may not be representative of students in other types of institutions or different geographical regions. Future research should test the model’s robustness with broader, more diverse samples. In addition to addressing these limitations, future studies could expand the current model by including other psychological traits, such as self-efficacy (Dai et al., 2025), to provide a more comprehensive understanding of the mechanisms linking policy to behavior.

## 5 Conclusion

This study identified significant gender differences in physical education policy recognition, time management ability, grit, and physical activity behavior among college students, with females scoring consistently lower. Mediation analysis revealed that school physical education policies indirectly influenced physical activity through time management ability, with grit significantly moderating this pathway. These findings underscore the critical role of psychological resources in transforming policy into behavior, and provide a theoretical foundation for optimizing physical education strategies in higher education settings.

## Data Availability

The original contributions presented in the study are included in the article/supplementary material, further inquiries can be directed to the corresponding author.
